# Foxi2 Is an Animally Localized Maternal mRNA in *Xenopus*, and an Activator of the Zygotic Ectoderm Activator Foxi1e

**DOI:** 10.1371/journal.pone.0041782

**Published:** 2012-07-27

**Authors:** Sang-Wook Cha, Meredith McAdams, Jay Kormish, Christopher Wylie, Matthew Kofron

**Affiliations:** 1 Cincinnati Children's Hospital Medical Center, Cincinnati, Ohio, United States of America; 2 University of Calgary, Calgary, Alberta, Canada; University of Colorado, Boulder, United States of America

## Abstract

Foxi1e is a zygotic transcription factor that is essential for the expression of early ectodermal genes. It is expressed in a highly specific pattern, only in the deep cell layers of the animal hemisphere, and in a mosaic pattern in which expressing cells are interspersed with non-expressing cells. Previous work has shown that several signals in the blastula control this expression pattern, including nodals, the TGFβ family member Vg1, and Notch. However, these are all inhibitory, which raises the question of what activates Foxi1e. In this work, we show that a related Forkhead family protein, Foxi2, is a maternal activator of Foxi1e. *Foxi2* mRNA is maternally encoded, and highly enriched in animal hemisphere cells of the blastula. ChIP assays show that it acts directly on upstream regulatory elements of Foxi1e. Its effect is specific, since animal cells depleted of Foxi2 are able to respond normally to mesoderm inducing signals from vegetal cells. Foxi2 thus acts as a link between the oocyte and the early pathway to ectoderm, in a similar fashion to the vegetally localized VegT acts to initiate endoderm and mesoderm formation.

## Introduction

One of the first, and major, patterning events in all triploblastic embryos is the formation of the three primary germ layers. In the early *Xenopus* embryo, the endoderm germ layer is specified by maternally encoded VegT [1] a T-box transcription factor that is localized to the vegetal cytoplasm in the oocyte, and inherited by the vegetal cells of the blastula [2], [3], [4]. As well as activating endoderm-specifying genes, VegT also activates expression of members of the nodal family of signaling ligands, that induce mesoderm to form in the adjacent equatorial region of the blastula [5], [6]. Thus, a single transcription factor can play an essential role in the initiation of two primary germ layers.

Much less is known about the formation of the ectoderm, which arises from the most animally located cells of the blastula [7], [8]. At the mid-blastula stage, these cells are pluripotent, as defined by their ability to form derivatives of different germ layers when transplanted to other regions of the blastula [9], and their ability to form mesoderm when cultured in combination with vegetal cells [10], [11] or by added soluble mesoderm inducers [12]. However, by the early gastrula stage, some three hours later at 21°C, animal cells no longer enter other lineages when transplanted [9], and no longer respond to mesoderm inducing signals [13], [14].

Once specified, the ectoderm cells spread to cover the entire surface of the embryo during gastrulation, before segregating into neural ectoderm that invaginates to form the central nervous system (CNS) and non-neural ectoderm that spreads over the entire surface of the embryo and differentiates into epidermis. Little is known about the initiation of this process, and we set out to test the hypothesis that the ectoderm is initially specified by maternal activator(s), as previously shown for the endoderm. In an effort to identify early zygotic genes whose expression might be activated throughout the ectoderm (both neural and non-neural), and thus targets of putative maternal activator(s), we compared array databases made from control embryos and embryos depleted of VegT, and from animal and vegetal cells at the early gastrula stage. The early zygotic gene whose expression was most up-regulated in both comparisons was *Foxi1e*, an i-class Forkhead type transcription factor [15]. This gene was already known to be expressed in animal cells [16], and its function was thought to be to repress mesoderm formation. We showed by both gain and loss of function, that, in fact, Foxi1e is an activator of both neural and epidermal genes, and is thus the first known early zygotic pan-ectoderm activating gene [15].

Expression of Foxi1e occurs in the animal hemisphere during the time of ectoderm specification; the mid blastula to mid-gastrula stage, and its expression pattern is complex. Foxi1e mRNA appears first dorsally, and spreads to the whole animal hemisphere by the mid-gastrula stage. Expression is then lost dorsally, so that by the early neurula stage, Foxi1e expression is confined to the non-neural ectoderm (the presumptive epidermis). Throughout its expression, *Foxi1e* mRNA is enriched in deep, compared to superficial cells of the ectoderm, and is mosaic; with Foxi1e-expressing cells interspersed with non-expressing cells [17]. Both long and short range signals control the complex expression pattern of Foxi1e. Loss of signaling through the Notch pathway, the nodals downstream of VegT, or through the maternal TGF-β family member Vg1, all cause up-regulation of *Foxi1e* mRNA, and loss of its mosaic pattern of expression. However, expression does not spread into the superficial cells, nor into the vegetal hemisphere. Clearly there are more controls remaining to be identified, particularly as all of the signals so far identified in the blastula that control expression of Foxi1e are repressors. This raises the major question of what activates its expression in the animal hemisphere. We hypothesized that the final expression pattern of Foxi1e is determined by a combination of maternally encoded activators and regional repressors in the blastula. To test this, and to identify putative maternal activators of Foxi1e, we analyzed the 5 kb upstream sequence of *Xenopus tropicalis Foxi1e* from the JGI (Joint Genome Institute) sequencing project, cloned and sequenced the 3.5 kb upstream sequence of the *Xenopus laevis Foxi1e* gene, and compared and scanned the sequences for common transcription factor binding sites. We then assayed EST databases for candidate transcription factors that are maternal, and whose mRNAs are concentrated in the animal hemisphere of the oocyte, and are therefore inherited at highest concentration by animal cells. We report here that another Forkhead family member, Foxi2, whose mRNA is inherited from the egg [18], is highly enriched in animal cells of the blastula, and is an essential activator of Foxi1e. Foxi2 thus provides the link between the maternal mRNA stockpile and the formation of the ectoderm, as does VegT for the endoderm.

## Results

### *Foxi2* mRNA and protein are expressed maternally and localized to the animal half of the *Xenopus* oocyte and embryo

**Figure 1:**
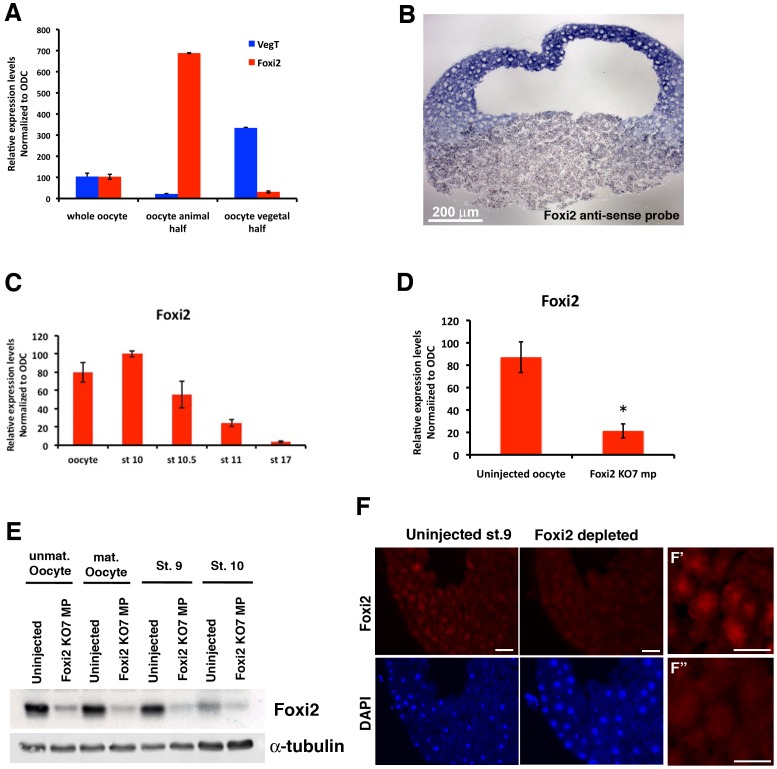
The spatial and temporal expression pattern of Foxi2 and its depletion at blastula stage. (A) RT-PCR to show relative expression levels of Foxi2 and VegT in either whole oocytes or dissected animal and vegetal halves. Foxi2 and VegT have reciprocal expression domains, with Foxi2 being highly enriched in the animal hemisphere. (B) In situ hybridization of tissue section at the late blastula stage to show distribution of *Foxi2* mRNA. (C) RT-PCR analysis of developmental time course to show absence of early zygotic expression of *Foxi2*. (D) RT-PCR to show degree of depletion of *Foxi2* mRNA by antisense oligo. (E–F) Degree of depletion of Foxi2 protein by mRNA depletion; a western blot of oocytes and early stages is shown in (E), and by immunostaining at the late blastula stage in (F). Scale bar in (F) = 50 µm. High magnification Foxi2 immunostaining images from Uninjected (F′) and Foxi2 depleted (F″) embryos. Scale bars in F′ and F″ = 20 µm.

Depletion of VegT was previously shown to cause a dramatic up-regulation of Foxi1e expression in the animal hemisphere, and a much smaller one in the vegetal mass [15]. This suggested the existence of a maternal activator(s) of ectoderm formation that is enriched in the animal hemisphere. **Figure 1** shows by real-time RT-PCR (**Figure 1A**) and in situ hybridization (**Figure 1B**) that *Foxi2* mRNA is expressed in the oocyte, enriched in the animal hemisphere, and is expressed in all cells of the animal hemisphere at the late blastula stage (the period of ectoderm specification). It is interesting to note that the mRNA has a gradient of concentration from the animal pole to the equator of the embryo. **Figure 1C** shows the temporal expression of *Foxi2* during early *Xenopus* development. mRNA levels decrease dramatically after gastrulation, showing that there is no zygotic expression, and therefore the major function of this mRNA must be in the initiation of zygotic transcription. In order to perform loss-of-function experiments for *Foxi2*, we screened antisense oligos to target both pseudoallelic maternal mRNAs in oocytes. **Figure 1D** shows the depletion of *Foxi2* mRNA using the KO7 oligo, which is complementary to nucleotides 191–208 of *Foxi2a* (AJ868112) and nucleotides 313–330 of *Foxi2b* (BC082945) of these maternal mRNAs. Approximately 80% of the mRNA was removed by a single 3 ng dose of KO7 oligo. The primers used to detect *Foxi2* in real-time RT PCR detected both *Foxi2a* and *Foxi2b*. After fertilization of Foxi2-depleted oocytes, the mRNA was not replaced at the onset of zygotic transcription (not shown), indicating that there is no zygotic activation of *Foxi2* in the embryo. In order to determine the effect of maternal mRNA depletion on Foxi2 protein levels at the blastula stage, a rabbit antibody (AB50) was raised against the C-terminal peptide CTSVMNPFGLNHLYSREGEV. This sequence is encoded by both pseudoalleles. **Figure 1E** shows by western blotting the degree of reduction of the Foxi2 protein at the late blastula and early gastrula stages. In the wild type, Foxi2 protein was present in matured *Xenopus* oocytes, and levels decreased slightly by the late blastula stage and more dramatically by the early gastrula stage. Foxi2-depleted oocytes and embryos show significant reductions of Foxi2 protein. Immunocytochemistry at the late blastula stage showed that Foxi2 protein was concentrated in cell nuclei, and enriched animally, as suggested by the distribution of the mRNA. In late blastulae derived from Foxi2-depleted oocytes there was a significant reduction in Foxi2 protein in cell nuclei, which are counterstained with DAPI in **Figure 1F**.

### Foxi1e Expression is reduced in Foxi2 depleted embryos and explants

**Figure 2:**
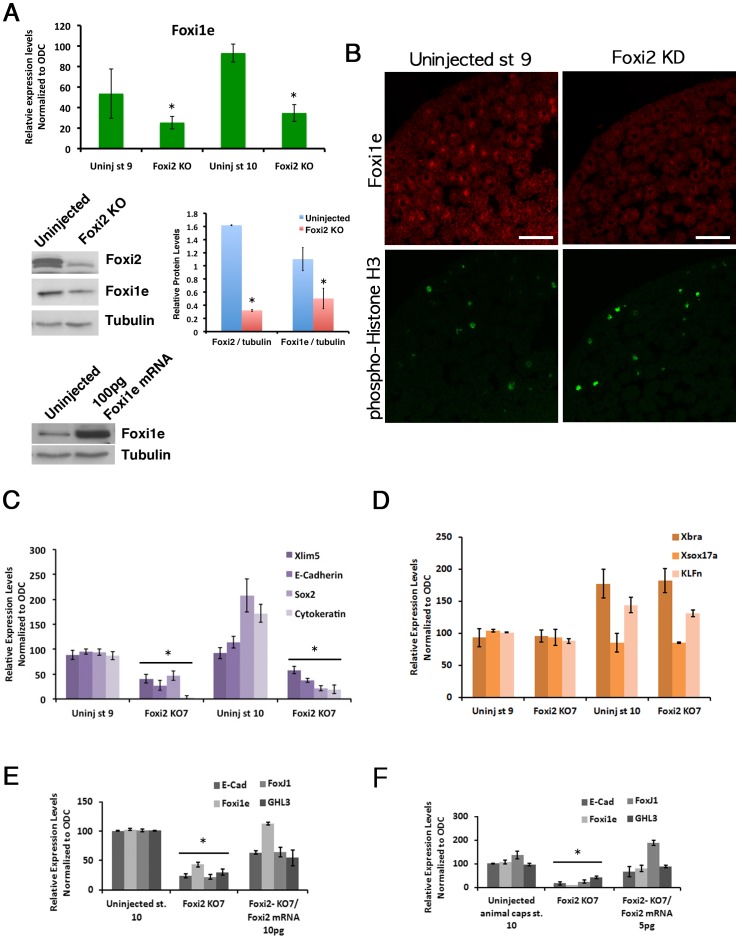
Expression of Foxi1e, as well as early ectodermal markers, requires Foxi2. (A–C) Show effect of Foxi2 depletion of *Foxi1e* mRNA levels at the late blastula and early gastrula stages (A), and on Foxi1e protein levels by western and by densitometric analysis of the western blot (A: middle panels) and immunostaining (B). (A) also shows by western blotting that the anti-Foxi1e antibody cross-reacts with *Xenopus* Foxi1e protein (lower panel). (C) Shows by RT-PCR the reduced levels of expression of ectodermal genes including *Xlim5*, *E-cadherin*, *Sox2* and *Cytokeratin* in control and Foxi2-depleted embryos at the late blastula and early gastrula stages. (D) RT-PCR shows that expression levels of the mesodermal marker *Xbra*, endodermal marker *Xsox17a* and ectodermal marker, *KLFn* are unaffected by Foxi2 depletion. (E–F) show that reduction in expression of the ectoderm marker genes *E-cadherin* (E-cad), *Foxi1e*, *FoxJ1*, and *grainyhead-like 3* (GHL3) caused by Foxi2 depletion are rescued by subsequent injection of *Foxi2* mRNA (10 pg dose) in both whole embryos (E) and animal caps (F). Scale bar in (B) = 50 µm.

We next tested whether Foxi2 is a maternal activator of Foxi1e expression. **Figure 2A** shows real-time RT-PCR analysis of control and Foxi2 depleted late blastula and early gastrula stage embryos. Foxi1e expression is reduced to 40% of control levels. In order to assay the effects on Foxi1e protein levels, we raised an antibody (AB98) against the N-terminal peptide sequence CESFLHPQTMPSPQRPSNFETGD in Foxi1e. The specificity of the antibody was verified by western blotting of embryos injected with 100 pg *Foxi1e* mRNA (**Figure 2A** **, lower panel**). This panel also shows the reduction in both Foxi2 protein, and Foxi1e protein, in Foxi2-depleted late blastulae. **Figure 2B** shows by immunostaining the degree of reduction of Foxi1e protein in blastula. The uninjected sibling embryos show the mosaic staining pattern corresponding to the previously documented mRNA expression pattern [6]. These sections were also stained with an antibody against phospho-histone H3(PH3), to identify dividing cells, in order to test the possibility that the mosaic pattern of Foxi1e expression is due to its presence only during (or between) cell divisions. Analysis of Foxi1e and PH3 staining patterns showed the presence of cells with either or both of the proteins present (28% of Foxi1e positive cells were also positive for PH3 at late blastula stage (98 cells from 12 individual sections were scored at the late blastula stage), indicating that the expression of Foxi1e in a mosaic pattern is not due to cell cycle-dependent expression. We then assayed the levels of expression of other markers of primary germ layer formation by real-time RT-PCR. **Figure 2C** shows expression of the neural marker *Sox2*, the epidermal markers, *Xlim5*, *E-cadherin* and *Cytokeratin* which are expressed in the animal hemisphere. These markers are highly up-regulated in VegT-depleted embryos. Expressions of *Sox2*, *E-cadherin* and *Cytokeratin* were reduced at the late-blastula and gastrula stages in Foxi2 depleted embryos. However, another ectodermal marker KLFn (*Xenopus* Neptune) was not affected, indicating that Foxi2 is not a pan-ectodermal activator (**Figure 2D**). We also looked at expression of markers of mesoderm and endoderm formation, **Figure 2D** shows that expression of both the general mesoderm marker *Xbra* and the endoderm marker *Sox17* were unaffected. The effect of Foxi2 oligo was specifically due to *Foxi2* mRNA depletion, as levels of expression of *Foxi1e* and *E-cad* were partially rescued, both in whole embryos (**Figure 2E**) and in isolated animal caps (**Figure 2F**) by injecting *Xenopus Foxi2* mRNA (10 pg) into Foxi2-depleted oocytes before maturation.

### Foxi2-depleted animal caps maintain their competence for mesoderm induction

**Figure 3:**
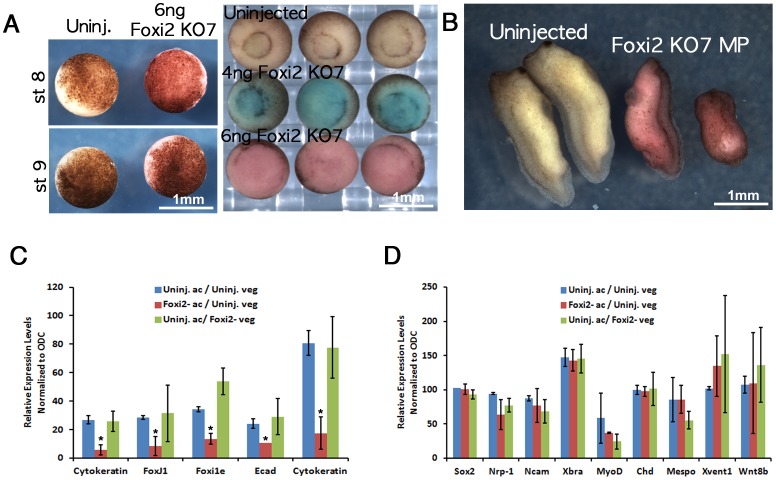
The phenotype of Foxi2-depleted (Foxi2 KO7) embryos. (A) Foxi2-depleted embryos were normal at the blastula stage (left panel) but showed highly delayed and incomplete gastrulation and 37/45 embryos showed major defects at the tail bud stage compared with controls, of which 25/26 were normal (right panel). (C) In Nieuwkoop recombinant assays, Foxi2-depleted animal caps expressed reduced levels of ectodermal markers. (D) Animal caps from Foxi2-depleted embryos express mesodermal markers in response to signals from vegetal masses.

In addition to the reduction of expression of ectodermal genes at the late blastula stage, Foxi2 depletion also caused delayed gastrulation movements, and subsequent major abnormalities to the embryo (**Figure 3A and B**). These were not investigated in detail here. However, we wished to assay the ability of the animal tissues of the blastula to form other germ layer derivatives if provided with appropriate signals, in order to exclude the possibility that Foxi2 depletion had a general transcriptional effect on animal cells. We therefore tested the ability of dissected animal caps to respond to mesoderm induction signals from the vegetal mass using Nieuwkoop recombinants. Animal caps from Foxi2-depleted embryos showed almost same expression levels of mesodermal markers as animal caps from uninjected embryos (**Figure 3C and 3D**), and Foxi2 depleted vegetal masses induced the same levels of mesoderm markers in control animal caps as did control vegetal masses (**Figure 3D**). These data show that the effect of Foxi2 depletion is specifically on ectoderm gene expression, and a general effect on animal cell transcription.

### Foxi2 is a direct maternal activator of Foxi1e expression, and binds the Foxi1e promoter

**Figure 4:**
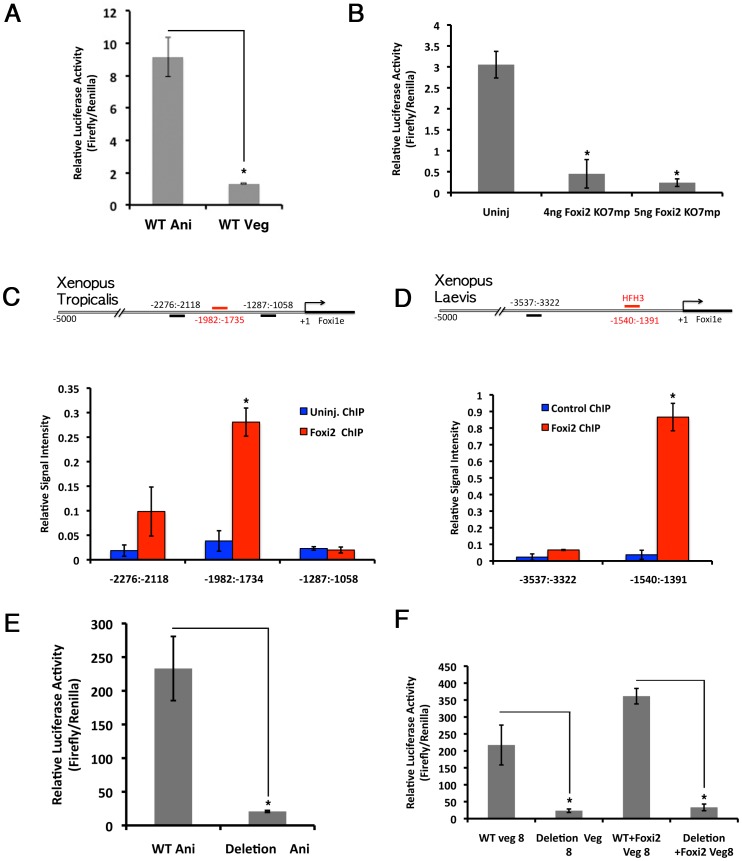
Foxi2 protein directly binds to the Foxi1e promoter. (A) Luciferase activity in blastulae after injecting *Xenopus* tropicalis *Foxi1e* promoter-luciferase construct into either animal or vegetal blastomeres at 8-cell stage embryos. (B) Foxi1e promoter activity was measured in blastulae derived from control (uninj) or Foxi2-depleted (4 ng and 5 ng) oocytes. (C,D) Chromatin immunoprecipitation assay. (C) A schematic diagram of *Xenopus* tropicalis Foxi1e promoter shows potential Foxi2 binding sites and ChIP PCR primer amplicons. Real-time PCR shows significantly higher signal on primer pair −1982∶−1734 after immunoprecipation by 6-myc-tagged Foxi2 protein. (D) A schematic diagram of *Xenopus* laevis Foxi1e promoter shows potential Foxi2 binding sites and ChIP PCR primer amplicons. Real-time PCR shows significantly higher signal on primer pair −1540∶−1391 after immunoprecipation by myc-tagged Foxi2 protein. (E) Luciferase activity in blastulae after injecting either wild-type (WT) *Xenopus* tropicalis Foxi1e promoter constructs or a construct lacking the Foxi2 binding region (Deletion). While wild-type promoter showed its activity (WT Ani), the construct lacking the Foxi2 binding site mutated promoter showed a basal level of promoter activity (Deletion Ani). (F) Luciferase activity in blastulae after injecting Wild-type and Deletion *Xenopus tropicalis Foxi1e* promoter constructs into vegetal blastomeres. *Foxi1e* promoter construct lacking the Foxi2 binding site showed a basal level of promoter activity (Deletion Veg 8) while Foxi2 overexpression in vegetal blastomeres had no effect on this mutated construct (Deletion+Foxi2 Veg 8) while wild-type promoter showed increased luciferase activity upon overexpression of Foxi2 (WT+Foxi2 Veg 8).

First, we cloned a 5 kb region identified *in silico* upstream of the transcription initiation site of *Xenopus tropicalis Foxi1e*. TRANSFAC analysis identified 4 good matches for Foxi class (HFH3/XFD2) binding sites in this 5 kb region ([19], [20], [21]. We subcloned the 5 kb sequence into pGL3 basic luciferase vector, and asked whether Foxi2 could activate luciferase activity driven by this promoter. **Figure 4A** shows that injection into animal cells of the 8-cell stage embryo caused a 9-fold increase in expression of the reporter construct, compared to vegetal injection, showing the presence of endogenous activators of the Foxi1e promoter that are enriched in the animal hemisphere. We then asked what effect Foxi2 loss-of-function had on luciferase expression driven by this promoter. **Figure 4B** shows the result of injecting the *Foxi1e* 5 kb luciferase promoter into the animal hemispheres of control and Foxi2-depleted embryos. Depletion of Foxi2 caused a 6-fold decrease in expression of luciferase driven by the Foxi1e 5 kb promoter at the early gastrula stage. We also tested whether this reported could be repressed by previously identified influences on Foxi1e [17]. Figure S1 shows that the reporter construct is responsive to the influence of Notch signaling but is not responsive to perturbations in nodal signaling. To test which region of the promoter had the greatest activity, we performed a stepwise deletion series from the 5′end of the construct (data not shown). We identified a region containing repressive elements at −4000 to −5000 from the initiation of transcription, and a strong activating domain at −1500 to −3000. (**Figure 4C**, A schematic diagram of *Xenopus tropicalis Foxi1e* promoter shows potential Foxi2 binding sites and ChIP primers' amplicons). To test whether Foxi2 binds directly to the upstream 5 kb sequence of *Foxi1e*, we performed chromatin immunoprecipitation experiments using *Xenopus* tropicalis embryos at the late blastula stage. In these experiments, we injected a sub-phenotypic dose of myc-tagged *Foxi2* mRNA (6-mycs, tandemly repeated) into the animal hemispheres of 2-cell stage embryos. We then immunoprecipitated the embryo lysates, using an antibody against myc, and analyzed the precipitated DNA by real-time PCR with primers specific to regions of *Xenopus* tropicalis *Foxi1e* 5 kb upstream sequence that contained predicted Foxi binding sites. The uninjected embryos served as a control. The strongest signal was seen in region −1982∶−1735 at 0.27% of input (**Figure 4C**). This was a 10-fold increase over the signal seen in ChIP DNA from uninjected embryos. An amplicon for an adjacent region, between nt −2276∶−2118 gave a weak signal by ChIP (0.1% of input). All other regions tested from this promoter gave signals less than or equal to background levels (−1287∶−1058 is shown). To further test whether Foxi2 could directly bind to the *Foxi1e* promoter, we cloned the Foxi1e promoter from *Xenopus laevis*. We compared the *Xenopus laevis* and *Xenopus tropicalis* promoters using CONSITE software and identified regions of strong conservation as well as potential Foxi binding sites. Chromatin precipitation experiments were performed identically as above. Real-time PCR was performed on ChIP DNA using primers flanking the potential Foxi binding sites. The region of the *Xenopus laevis Foxi1e* promoter that was orthologous to the ChIP positive region of *Xenopus tropicalis Foxi1e* gave a positive signal at 0.85% of input DNA (**Figure 4D**). All other regions of the promoter gave only background levels of signal by real-time PCR. We then asked if removal of the Foxi2 binding region would eliminate activity of the full-length *Foxi1e* promoter. A deletion of the Chip positive region (−1982∶−1735) was made in the *Xt Foxi1e-*Luc construct. The result of this deletion was the abrogation of 90% of the luciferase activity of the control construct in gastrulae derived from embryos injected into animal blastomeres of 4-cell stage embryos (**Figure 4E**). We also tested whether Foxi2 overexpression could activate expression from the deletion construct. Either the full-length or the deletion *Foxi1e*-luc construct was injected into vegetal cells of 8-cell stage embryos ± *Foxi2* RNA. Whereas *Foxi2* RNA activated luciferase expression from the full-length construct by about 2 fold, no activation was seen from the deletion construct (**Figure 4F**).

### Foxi2 is essential for other major signaling inputs on Foxi1e expression

**Figure 5:**
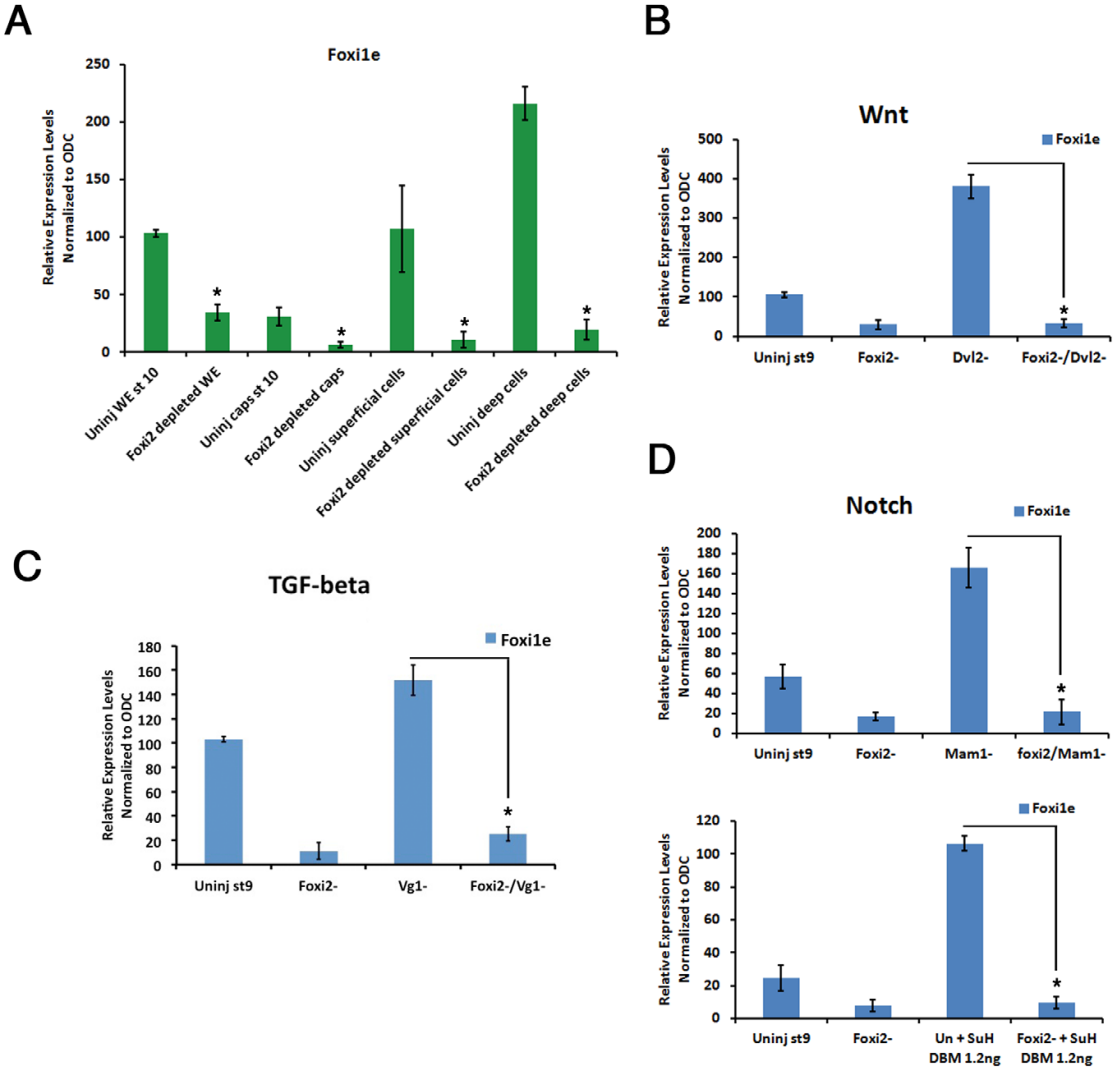
Spatial expression of Foxi1e is dependent on Foxi2. (A) The level of expression of Foxi1e is reduced in Foxi2-depleted animal caps (Foxi2 depleted caps) compared to the caps from control siblings (Uninj caps st.10) at the gastrula stage. Both dissociated superficial and deep cells from Foxi2-depleted embryos expressed reduced levels of *Foxi1e* mRNA. Note that the expression level of Foxi1e in dissociated superficial cells is much higher than in intact animal caps (Uninj superficial cells). (B) Maternal depletion of Dishevelled2 (Dvl2−) showed upregulation of Foxi1e expression. Double depletion of Foxi2 and Dvl2 (Foxi2−/Dvl2−) showed similar level of expression to Foxi2-depleted embryos (Foxi2−). (C) Maternal depletion of Vg1 (Vg1−) showed upregulation of Foxi1e expression. Double depletion of Foxi2 and Vg1 (Foxi2−/Vg1−) showed a level of expression similar to Foxi2-depleted embryos (Foxi2−). (D) Upper panel; Maternal depletion of transcription factor Mastermind1 (Mam1−) caused upregulation of Foxi1e expression. Double depletion of Foxi2 and Mastermind1 (Foxi2−/Mam1−) showed a similar level of expression as Foxi2 depleted embryos (Foxi2−). Lower panel; The overexpression of Supressor of Hairless (SuH) DNA binding mutant (SuH DBM) mRNA caused upregulation of Foxi1e expression. Foxi1e expression in Foxi2 depleted+1.2 ng of *SuH DBM* mRNA injected embryos showed similar level of expression as Foxi2 depleted embryos (Foxi2−).

We have shown previously that intercellular signals in the blastula, including Notch, nodal, and Vg1, all repress Foxi1e expression, and removal of these signals causes Foxi1e expression in all cells of its expression domain (the deep cells of the animal hemisphere), instead of in the normal mosaic pattern [17]. To test the role of Foxi2 in this up-regulation, we dissected animal caps from control and Foxi2-depleted mid-blastulae. These were either cultured intact, or separated into deep cells (which normally express Foxi1e) and superficial cells (which do not). These two cell populations were dissociated in calcium/magnesium-free saline. The effect of this is to remove both long range signals from other regions of the embryo, and short range (for example Notch) signals from adjacent cells. We hypothesized that if Foxi2 is a maternal activator of Foxi1e, then its depletion should block the resulting increase in Foxi1e expression. This proved to be the case. **Figure 5A** shows the real-time RT-PCR analysis of this experiment. There was a dramatic rise in Foxi1e expression in both superficial and deep animal cells when they were dissociated, which was completely blocked by *Foxi2* mRNA depletion.

This experiment eliminated all intercellular signals. To test each pathway directly, we blockaded Wnt signaling by depletion of maternal Dvl2 (**Figure 5B**), Vg1 signaling by depleting maternal Vg1 (**Figure 5C**), and Notch signaling by depletion of maternal mastermind (NM_001087458) (**Figure 5D** **, upper panel**) or by injection of a mutated SuH mRNA (**Figure 5D** **, lower panel**). In each case the up-regulation of Foxi1e expression caused by a blockade of these repressors was abrogated by depletion of maternal *Foxi2* mRNA. These data show that Foxi2 is an essential activator of Foxi1e, and that this activation is repressed by intercellular signaling in the blastula, which confines expression of Foxi1e to its mosaic expression in the deep animal cells.

## Discussion

Forkhead genes, originally identified in Drosophila [22], are represented in the genomes of animal species (though not plants), from yeast to man. The DNA-binding Forkhead domain is highly conserved, but there is wide sequence divergence outside this domain, giving rise to 35 families of Fox genes in humans and mice. Fox genes play essential roles in development and differentiation, the immune system, the cell cycle and cancer, in species longevity, and metabolism. Mutations in Fox genes cause many human congenital disorders [23].

The *Foxi* class is still poorly understood. So far only identified in deutostomes, expression patterns, and some functional data have been published for *Ciona* [24], *Xenopus* [18], [25], [26], [27], Zebrafish [28], and mouse [29], [30]. All Foxi genes for which expression patterns have been published show some expression in the ectoderm, as well as other tissues, although early expression patterns corresponding to the times described here for *Xenopus* have not been well-studied. In the mouse and Zebrafish, *Foxi1* is expressed in the otic placodes and structures derived from them (the endolymphatic duct in the mouse for example), and mutations of *Foxi1* in both species cause defects in sensory structures derived from the otic placodes [28], [29]. *Foxi2* in the mouse is also expressed in ectodermal structures, including olfactory epithelium, whiskers, dental epithelium and otic placode [23], [31]. *Foxi3* in the mouse is expressed in an ectodermal region defined as pan-placodal, as well as hair follicles and dental epithelium [31], [32]. Furthermore, it has recently been shown that the loss of hair and teeth in Mexican and Peruvan hairless dogs (canine ectodermal dysplasia) is caused by a mutation in the *Foxi3* gene [32], confirming a role for this gene in ectodermal differentiation. In Zebrafish, *Foxi3a* and *b* are expressed in early ectoderm (Solomon et al. 2003). In Ciona, three Foxi class genes have been identified (*FoxIa-c*), derived from duplications independent of those identified in vertebrates [33].

Two Foxi class members; Foxi2 and Foxm1 are expressed maternally in *Xenopus* [34], whilst Foxi1e has been shown to be expressed zygotically in the animal hemisphere at the blastula stage [15], [16]. Foxi1e represses the response to mesoderm induction in animal cells [16] and activates transcription of ectodermal genes [15]. Foxi1e expression during the late blastula and early gastrula stages is highly dynamic and becomes localized by the mid-gastrula stage to a mosaic pattern in which expressing cells are mixed with non-expressing cells in the deep cell layers of the animal hemisphere. This expression domain is apparently controlled by long and short-range signals, including Notch, nodals, and the TGFβ family member Vg1 [17]. Here we show that Foxi2, expressed in the egg, where it is enriched in the animal hemisphere, is a direct activator of Foxi1e expression at the blastula stage.

It is interesting that Foxi2 is expressed as an apparent gradient of mRNA from the animal pole to the equator of the blastula. It was not possible to tell from immunostaining whether the protein concentration is also patterned similarly. This will have to await a more sensitive immunocytochemical assay. It is also difficult to generate, by mRNA depletion and replacement by different doses of injected mRNA, functionally different physiologically relevant levels of *Foxi2* mRNA. It has to be remembered also, that the amount of yolk-free cytoplasm decreases from animal to vegetal, so all mRNAs will decrease in apparent concentration along this axis, unless they are (like Veg1 and VegT) specifically localized vegetally.

This work shows that, with respect to maternal transcriptional activators, the *Xenopus* egg is bipolar, with VegT concentrated vegetally and Foxi2 concentrated animally. Although these two transcription factors do not, as far as is known, repress each others' action directly, they do so by proxy. VegT initiates expression of nodals, which activate mesoderm and inhibit Foxi1e expression, whilst Foxi2 initiates expression of Foxi1e, which activates ectoderm genes and represses mesoderm. Although these are, almost certainly, not all of the maternal primary germ layer determinants, they show how the formation of three germ layers can be generated along the animal-vegetal axis of the blastula.

### Ethics Statement

All *Xenopus* laevis experiments in this study have been conducted under protocol # 2D02014, approved by the Cincinnati Children's Research Foundation's Institutional Animal Care and Use Committee.

## Materials and Methods

### Oocytes and embryos

All *Xenopus* animals were purchased from Nasco (Fort Akinson, WI). Host transfer experiments were done following the protocol described previously [35]. All antisense oligos were injected into isolated oocytes in the equatorial region and cultured for at least 48 hours before injecting various mRNA constructs as described in the text. In general, embryos were cultured in 0.1× MMR.

### DNA constructs and mRNA

Full-length *Xenopus tropicalis Foxi2* cDNA was acquired from the *X. tropicalis* unigene library (clone TGas144f13) in pCS107 vector. *Foxi2* mRNA was synthesized after linearizing with Asp718 digestion using the SP6 mMESSAGE mMACHINE kit (Ambion). 6-myc tagged *Xl. Foxi2* was cloned by addition of a 5′ EcoR1 site by proofreading PCR amplification. This site allowed us to subclone the CDS of *Foxi2* in-frame with 6 N-terminal myc tags from pCS2 −6 myc. 6-myc-*Foxi2* mRNA was synthesized after linearizing with NotI digestion using the SP6 mMESSAGE mMACHINE kit (Ambion). This *Su(H) DBM* mRNA The plasmid for synthesizing the *Su(H)DBM* RNA was a gift from Chris Kintner [36]. pCS2 Xl Su(H)DBM was linearized with NotI and transcribed using the SP6 mMEESAGE mMACHINE kit (Ambion).

### Oligonucleotides

Anti-sense oligos are the following: Foxi2 KO7, 5′ G*A*G*CTGCTGGTCGCT*G*A*G 3′ was used at 3.5 ng per oocyte. Mam1 AS-2, 5′ C*G*C*GTGCATCCGCTC*C*T*C 3′ was used at 5 ng per oocyte. *Indicates a phosphorothiate modified bond. Vg1 and Dvl oligos used as previously described [37], [38].

### Quantitative RT-PCR and in situ hybridization

Total RNA from oocytes, explants and early embryos was isolated using the protocol of Mir et al. (2007). Real-time RT-PCR was performed using a LightCycler (Roche). Water-blank and RT-minus controls were included in all runs. All RT-PCR results are presented as percentage compared with the level in uninjected embryos after normalization to the expression of ornithine decarboxylase (ODC). The graphs in figures show means ± S.D. Statistical analysis has been performed on all RT-PCR data and marked on the graphs with asterisk (*) when p-valve is less than 0.05 when comparing control and experimental groups by two-tailed student's t-test. Whole-mount in situ hybridization was as described [38].

### Western blot

Western blots were carried out as described previously [38]. Antibody concentrations were: rabbit anti-Foxi2 (1∶1000 dilution), guinea pig anti-Foxi1e (1∶500 dilution) and mouse anti-tubulin (DM1A, Neomarker), in 1∶5000 dilution. For quantitation of protein bands, ImageJ software was used.

### Tissue manipulation

For Nieuwkoop recombinant assay, *Xenopus* embryos obtained by host transfer method were maintained in 0.1× MMR medium. At stage 8, vegetal explants were dissected in 1× MMR medium and allowed to heal for 10 minutes. Animal caps were cut and placed on top of vegetal explants in 2% Agarose dish for 2 hours before being processed by RT-PCR.

For animal cap dissociation experiments, Animal caps were removed from blastulae at about stage 8.5, and their cells dissociated by incubation in phosphate buffer for 2 mins and transfer to CMFM for 10 minutes. Deep Cells were dispersed by gentle pipetting and the pigmented superficial layer transferred back in phosphate buffer for 10 mins to achieve its complete dissociation. Dissociated pigmented cells were collected and transferred to CMFM for further incubation. After 2 hrs incubation, cells were collected for RT-PCR.

### Luciferase assay

Various Foxi1e promoter constructs were injected together with 25 pg pRLTK DNA as described in text. Three replicate samples each of three embryos were frozen for each group at the early blastula stage and assayed using Promega Dual-luciferase assay system.

### ChIP assay

Chromatin immunoprecipitation (ChIP) was carried out with *Xenopus* embryonic tissues essentially as in [39]. Groups of 50 embryos were fixed at stage 10 in 1% formaldehyde in 1× PBS for 10 minutes. The fixation reaction was quenched with ice-cold 125 mM glycine in 1× PBS for 10 minutes. Embryos were then homogenized in ice-cold nuclear lysis buffer (20 mM Tris-HCl[pH 8.0], 60 mM KCl, 13 mM NaCl, 1 mM EDTA, 1 mM EGTA, 1 mM DTT, 0.5 mM PMSF, 1 mM Benzamidine, 5 µg/mL antipain, 5 µg/mL leupeptin, 5 µg/mL trypsin inhibitor). Lysates were flash-frozen in liquid nitrogen and stored at −80°C. Lysates were cleared and sonicated to fragment the genomic DNA into average size of 500 bp. Sonicated DNA was incubated with a rabbit anti-myc antibody (Sigma C3956) and immunoprecipitated with protein A beads. Crosslinking was reversed, and DNA was purified by phenol/chloroform extraction followed by precipitation. Real-time PCR was performed on this DNA with primers from multiple regions of the *Xenopus tropicalis* and *laevis* Foxi1e promoters.

Xt primer pair −2276∶−2118 −2276-U-5′-TGAGGAAAGGGTCTGAGAGAA-3′, -2118-D-CAGCAATCCATTATGCCTCA-3′

Xt primer pair −1982∶−1734 −1982-U-5′-GGAGGCTGGAGACGTTGATA-3′, -1734-D-GCTATTCATGCAGGGACCAG

Xt primer pair −1287∶−1058 −1287-5′-TAACGGGTTTCCGGATAAGA-3′, -1058-U-5′-TACATCCCTGTGTTGCCTGT-3′

Xl primer pair −3557∶−3322 −3557-5′-U-CTTCCCAGCAATACCCCATA-3′, -3322-D-5′- ATGTCATCCATCCCACCTGT-3′

Xl primer pair −1540∶−1391 −1540-U-5′-CAGGCAGCACTCTTCATTCA-3′, -1391-D-5′-GCTGAGGGTAGTGCTTGTGC-3′

## Supporting Information

Figure S1 **Foxi1e promoter construct is responsive to perturbations of Notch signaling but is not responsive to the influence of Nodal signaling.** Either control (FL) or a construct lacking the Foxi2 binding region (Mut) *Xenopus* tropicalis *Foxi1e* promoter-luciferase constructs were injected into animal blastomeres of 8-cell stage embryos. When reporter-injected embryos are co-injected with mRNA encoding the Notch intracellular domain (NICD) to activate Notch signaling, there is a 5-fold reduction in luciferase activity of the wild-type promoter construct. Ectopic activation of Notch signaling also gives a 2.5-fold reduction in the activity of the promoter construct that lacks the Foxi2 binding domain (Mut). Foxi1e luciferase activity is unaffected by perturbing Nodal signaling via injection of Cerberus Short (CerS) mRNA.
(JPG)
