# Supplementary figures and images for: Foxi2 Is an Animally Localized Maternal mRNA in Xenopus, and an Activator of the Zygotic Ectoderm Activator Foxi1e
Source: PLoS One. 2012 Jul 27;7(7):e41782. doi: 10.1371/journal.pone.0041782 (PMC3407068; doi:10.1371/journal.pone.0041782)

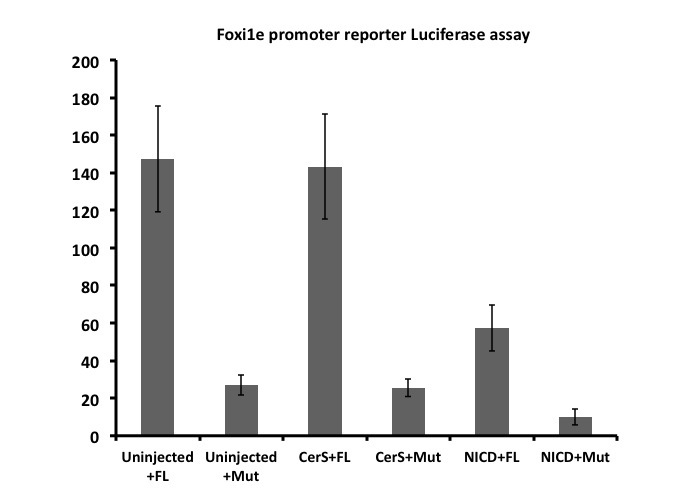

Supplement: Figure S1 — Foxi1e promoter construct is responsive to perturbations of Notch signaling but is not responsive to the influence of Nodal signaling. Either control (FL) or a construct lacking the Foxi2 binding region (Mut) Xenopus tropicalis Foxi1e promoter-luciferase constructs were injected into animal blastomeres of 8-cell stage embryos. When reporter-injected embryos are co-injected with mRNA encoding the Notch intracellular domain (NICD) to activate Notch signaling, there is a 5-fold reduction in luciferase activity of the wild-type promoter construct. Ectopic activation of Notch signaling also gives a 2.5-fold reduction in the activity of the promoter construct that lacks the Foxi2 binding domain (Mut). Foxi1e luciferase activity is unaffected by perturbing Nodal signaling via injection of Cerberus Short (CerS) mRNA. (JPG) [file pone.0041782.s001.jpg]
